# Rumination Swallowing in Limb-Girdle Muscular Dystrophy

**DOI:** 10.7759/cureus.68846

**Published:** 2024-09-07

**Authors:** Koji Hayashi, Shiho Mitsuhashi, Mamiko Sato, Yuka Nakaya, Asuka Suzuki, Yasutaka Kobayashi

**Affiliations:** 1 Department of Rehabilitation Medicine, Fukui General Hospital, Fukui, JPN; 2 Graduate School of Health Science, Fukui Health Science University, Fukui, JPN

**Keywords:** barium swallow study, dysphagia', evaluations for dysphagia, limb-girdle muscle dystrophy, rumination swallowing

## Abstract

Patients with limb-girdle muscular dystrophy (LGMD) may develop swallowing disorders as the disease progresses. We detected a novel swallowing pattern in patients with LGMD, characterized by unique findings such as a rumination-like behavior, where food reaches the vallecula and piriform sinuses and then is regurgitated back into the oral cavity, re-chewed, and swallowed again. We termed this swallowing pattern "rumination swallowing." In this report, we describe a case of rumination swallowing, as observed through a videofluoroscopic (VF) examination, in a 56-year-old patient with LGMD.

## Introduction

Limb-girdle muscular dystrophies (LGMD) constitute a group of disorders that primarily affect the skeletal muscles, particularly those in the hips and shoulders [1]. These conditions are caused by mutations in genes encoding specific proteins, unique to each subtype [1]. The inheritance pattern of LGMD is autosomal, with some forms being dominant and others recessive [1]. It has been reported that 30% of patients with LGMD experience some feeding-related abnormalities [2], including obvious dysphagia and radiographical or physiological abnormalities related to swallowing. In addition, among genetically confirmed LGMD patients, LGMD 2B and 1D have been reported to lead to dysphagia [3-5]. To the best of our knowledge, no studies related to LGMD have described swallowing disorders with reflux of the food bolus. We present a case of LGMD with a peculiar swallowing pattern characterized by repeated regurgitation of a food bolus, chewing it, and then swallowing it again, which we have termed "rumination swallowing."

## Case presentation

A 56-year-old Japanese male visited our hospital for a swallowing examination. He had developed walking difficulties at the age of 25 and had been diagnosed with LGMD by muscle biopsy at age 40. His older brother had also been diagnosed with LGMD and died at age 53. Although he had undergone genetic testing for muscular diseases, no known genetic abnormalities had been identified. He had developed breathing problems at age 42 and had been subsequently diagnosed with sleep apnea. Around the age of 53, he had begun using a manual wheelchair. At age 44, non-invasive positive-pressure ventilation (NPPV) had been applied due to an exacerbation of his breathing difficulties. Despite the adjustments made to NPPV, his dyspnea had continued to gradually progress.

Around age 46, he had developed dysphagia. At age 48, he had started using an electric wheelchair. At age 50, his activity of daily living (ADL) had further deteriorated due to respiratory and swallowing disorders. He had begun to experience choking on saliva, prolonged meal times, and regurgitation of food into the oral cavity. He explained that when he felt a sensation of food getting stuck in his throat while swallowing, he would intentionally regurgitate it into the oral cavity to make it easier to swallow. In addition, severe constipation and subileus had been occasionally noted, and he had visited the gastroenterology department several times. Around the age of 52, he had begun experiencing dyspnea while eating meals. By age 56, his ADLs had almost become entirely dependent on assistance, and he used a power wheelchair for mobility. Subsequently, he had been admitted for a swallowing evaluation, which included both fiberoptic endoscopic examination and videofluoroscopic (VF) examination.

Neurological examination on admission revealed normal cognition, grade three muscle strength in the bilateral sternocleidomastoid and trapezius muscles, grade two muscle strength in the bilateral proximal muscles of the upper limbs, grade four muscle strength in the distal muscles of the upper limbs, and grade one to two muscle strength in the bilateral proximal and distal muscles of the lower limbs and areflexia. In addition, joint contractures were observed in various joints. No ptosis, facial muscle weakness, sensory disturbance, or ataxia was observed. VF revealed that the swallowing pressure was weak, resulting in pharyngeal residue, forcing him to swallow the residual food multiple times. Additionally, swallowed liquid barium, semi-solid foods, and soft rice reaching the vallecula and piriform sinuses, and then regurgitating back into the oral cavity (Video 1) were observed. Then, the patient re-chewed the bolus and attempted to swallow, especially semi-solid food and soft rice (Video 2). This appearance resembled the rumination of a cow. During VF, he was aware of food regurgitation into the oral cavity. He was discharged from the hospital after receiving the results of this test.

**Video 1:** Videofluoroscopic (VF) examination with watery barium The videofluoroscopic (VF) examination revealed that, after being swallowed, the watery barium reached the vallecula and piriform sinuses, and then regurgitated back into the oral cavity

**Video 2:** Videofluoroscopic (VF) examination with soft rice The videofluoroscopic (VF) examination revealed that soft rice, after being swallowed, reached the vallecula and piriform sinuses, and then regurgitated back into the oral cavity, where the patient re-chewed the bolus and attempted to swallow again

## Discussion

We observed that swallowed liquids and foods were regurgitated from the pharynx into the oral cavity, chewed again, and then swallowed into the esophagus - a phenomenon we termed "rumination swallowing." We found two previous reports of swallowed material regurgitating back into the oral cavity in the literature. In the study by Bushmann et al., two out of 20 cases of Parkinson’s disease exhibited reflux of the bolus (puree and cookie) from the base of the tongue to the oral cavity in a repetitive manner [6]. The authors reported that no such phenomenon was observed with barium [6]. This report does not speculate on the mechanism behind the reflux of the food bolus into the oral cavity. Another study has reported the intraoral regurgitation of food boluses in patients with oculopharyngeal muscular dystrophy (OPMD) and dermatomyositis [7]. According to this report, solid and semi-solid foods were regurgitated into the mouth, but liquids were not regurgitated; instead, they were aspirated [7].

In the OPMD case, based on the patient's sensation of food residue in the piriform sinus and the awareness of the action of refluxing food into the oral cavity, the authors hypothesize that the patient may be deliberately returning the food bolus to the oral cavity due to impaired contraction of the pharyngeal muscles, which prevents the food bolus from being transported to the esophagus despite attempts at swallowing [7]. In contrast, in the dermatomyositis case, the patient was not aware of any reflux into the oral cavity and did not exhibit a swallowing reflex even when food boluses were retained in the epiglottic vallecula. Therefore, the authors speculate that the patient may be initiating a movement to reflux the bolus into the oral cavity and then subsequently reintroducing it into the pharynx to better synchronize the timing of the pharyngeal phase of swallowing [7].

Our patients had LGMD with unidentified genetic abnormalities. Therefore, it is difficult to compare him with patients who have identified types of muscular dystrophy. However, similar to other progressive muscular dystrophies, it is likely that dysphagia in these patients was due to muscle weakness in the pharyngeal and upper esophageal smooth muscles [8]. Our patient stated that he intentionally regurgitated foods and liquids into his mouth. This suggests that compensatory rumination swallowing may be acquired as an adaptation to dysphagia. Unlike previous reports, our case also demonstrated the regurgitation of liquids into the oral cavity, the reason for which remains unclear. This may be a compensatory ability that he uniquely acquired. However, it should be noted that because patients will regurgitate food forcefully and then attempt to swallow it, there is always a risk of aspiration pneumonia or choking. 

A unique swallowing method, vacuum swallowing, was reported in a study in 2018 [9]. This method is adopted after the onset of the disease, where patients create strong negative pressure in the esophagus to improve the passage of a bolus through the pharynx. This method has been reported in studies involving Wallenberg syndrome and spinal muscular atrophy (SMA) [9,10]. Interestingly, a patient with SMA can acquire vacuum swallowing through rehabilitation therapy. This suggests that vacuum swallowing is acquired as compensation for dysphagia, whether naturally or through training. Vacuum swallowing appears to move the bolus retained in the pharynx into the stomach by suctioning it into the esophagus, while rumination swallowing seems to compensate for weakness by repeatedly propelling the bolus from the mouth to the esophagus with force. We assumed that rumination swallowing may similarly serve as a compensatory mechanism for swallowing disorders. Further studies are needed to comprehend the mechanism behind rumination swallowing.

## Conclusions

We presented a case of rumination swallowing identified via VF in a patient with LGMD. Rumination swallowing is a method in which swallowed food is regurgitated from the pharynx into the oral cavity, chewed again, and then swallowed into the esophagus. This may be a compensatory mechanism for esophageal smooth muscle dysfunction. Further studies are needed to reveal the mechanism behind rumination swallowing.
